# Modeling sporadic juvenile ALS in iPSC-derived motor neurons explores the pathogenesis of FUS^R503fs^ mutation

**DOI:** 10.3389/fncel.2024.1364164

**Published:** 2024-04-22

**Authors:** Li Chen, Guojie Chen, Mengting Zhang, Xiaojie Zhang

**Affiliations:** ^1^Department of Neurology, Shanghai Sixth People's Hospital Affiliated to Shanghai Jiao Tong University School of Medicine, Shanghai, China; ^2^Shanghai Neurological Rare Disease Biobank and Precision Diagnostic Technical Service Platform, Shanghai, China; ^3^Hunan YoBon Biotechnology Limited Company, Changsha, Hunan, China; ^4^College of Integrated Chinese and Western Medicine (School of Life Sciences), Anhui University of Chinese Medicine, Hefei, Anhui, China

**Keywords:** juvenile amyotrophic lateral sclerosis, fused in sarcoma, physicochemical properties, neuroelectrophysiology, calcium activity imaging

## Abstract

**Introduction:**

Fused in sarcoma (FUS) mutations represent the most common genetic etiology of juvenile amyotrophic lateral sclerosis (JALS), for which effective treatments are lacking. In a prior report, we identified a novel FUS mutation, c.1509dupA: p. R503fs (FUSR503fs), in a sporadic JALS patient.

**Methods:**

The physicochemical properties and structure of FUSR503fs protein were analyzed by software: Multi-electrode array (MEA) assay, calcium activity imaging assay and transcriptome analysis were used to explore the pathophysiological mechanism of iPSC derived motor neurons.

**Results:**

Structural analysis and predictions regarding physical and chemical properties of this mutation suggest that the reduction of phosphorylation and glycosylation sites, along with alterations in the amino acid sequence, may contribute to abnormal FUS accumulation within the cytoplasm and nucleus of induced pluripotent stem cell– derived motor neurons (MNs). Multi-electrode array and calcium activity imaging indicate diminished spontaneous electrical and calcium activity signals in MNs harboring the FUS^R503fs^ mutation. Transcriptomic analysis reveals upregulation of genes associated with viral infection and downregulation of genes involved in neural function maintenance, such as the ATP6V1C2 gene. Treatment with ropinirole marginally mitigates the electrophysiological decline in FUS^R503fs^ MNs, suggesting the utility of this cell model for mechanistic exploration and drug screening.

**Discussion:**

iPSCs-derived motor neurons from JALS patients are promising tools for drug screening. The pathological changes in motor neurons of FUS^R503fs^ may occur earlier than in other known mutation types that have been reported.

## 1 Introduction

Amyotrophic lateral sclerosis (ALS) is a fatal neurological disorder characterized by progressive degeneration of motor neurons (MNs) in the corticospinal, bulbar, and spinal regions, ultimately resulting in paralysis and death within 3–5 years. The etiology of ALS is multifactorial, with over 25 known mutant genes contributing to approximately 70% of familial cases and 15% of sporadic cases (Goutman et al., 2023). Mutations in FUS are particularly associated with early-onset, aggressive forms of ALS. To date, more than 50 different FUS mutations have been identified in ALS patients, collectively accounting for approximately 4% of familial cases and 2% of sporadic ALS cases (Korobeynikov et al., 2022).

Fused in sarcoma (FUS) is a protein expressed ubiquitously, primarily located in the nucleus, with multiple functions in DNA damage repair and various aspects of RNA metabolism, including transcription, mRNA transport, pre-mRNA splicing, stability, and translation of microRNA and other non-coding RNAs (Dormann and Haass, 2013). It shuttles between the nucleus and cytoplasm, operating at a low cytoplasmic level under physiological conditions. However, in FUS-ALS patients (Kapeli et al., 2017), mutant FUS proteins mislocalize to the cytoplasm of MNs. The functional consequences of these ALS-associated mutations remain unclear. Evidence suggests that while loss-of-function alone is insufficient to cause MN death, toxic gain-of-function, including phenomena such as liquid–liquid phase separation (LLPS), stress granule (SG) formation, and involvement in the lysosomal autophagic system, contributes significantly (Scekic-Zahirovic et al., 2016). FUS’s nuclear import is facilitated by the C-terminal nuclear localization signal (NLS). Most ALS-causing mutations in FUS occur in this region, each displaying distinct molecular phenotypes. For example, arginine mutations impair RNA binding, while glycine mutations lead to rapid loss of fluidity (Niaki et al., 2020). Additionally, FUS-NLS mutations alter LLPS and SG associations, making FUS less sensitive to the chaperone activity of the nuclear import receptor transportin (TNPO1) (Hofweber et al., 2018). Previous reports suggest that the severity of the effect of FUS mutations on NLS function may correlate with the onset time and severity of ALS disease, as seen in variants such as p.R503fs in a 17-year-old juvenile amyotrophic lateral sclerosis (JALS) patient (Chen et al., 2020) and p.R495QfsX527 in a 19-year-old JALS patient (Belzil et al., 2012). Thus, understanding the structural and physicochemical properties of mutant FUS could guide laboratory research directions.

Our previous study identified a novel heterozygous FUS mutation, c.1509dupA:p.R503fs, in a 17-year-old female patient diagnosed with sporadic JALS who succumbed to the disease 15 months after onset (Chen et al., 2020). The early age of onset and rapid progression prompted further investigation. We observed increased FUS expression and suspicious accumulation in iPSC-derived MNs from the patient (Chen et al., 2020). However, the mechanism underlying JALS caused by the FUS^R503fs^ mutation warrants exploration. This mutation leads to the abnormal translation and early termination of the FUS protein in the NLS terminal, likely playing a pivotal role in disease development and progression. In this study, we explored the mutant FUS^R503fs^ extensively using bioinformatics analysis. We conducted electrophysiological tests on iPSC-derived MNs and examined their response to ropinirole treatment. Transcriptomic analysis revealed the downregulation of genes related to neural function maintenance, such as the autophagolysosomal-associated ATP6V1C2 gene. Ropinirole treatment partially alleviated the electrophysiological decline in FUS^R503fs^ MNs. Our findings suggest that the electrophysiological and spontaneous calcium activities of FUS^R503fs^ MNs are diminished, with ropinirole treatment showing slight improvement in some electrophysiological properties. Moreover, the decrease in spontaneous calcium activity signals may be independent of the glutamatergic receptor system, likely caused by other unknown mechanisms. The transcriptomic analysis also revealed the downregulation of genes related to neural function maintenance, along with the upregulation of genes associated with stress and viral infection. This study sheds light on the structural and physicochemical properties of this novel mutation and attempts to explore the pathophysiological characteristics of FUS-JALS in cell models, providing a basis and direction for further research into the pathological mechanisms.

## 2 Materials and methods

### 2.1 Participants and source of iPSC cell line

A 17-year-old Chinese girl was diagnosed with juvenile ALS, following the revised El Escorial diagnostic criteria. She carries a novel FUS mutation, c.1509dupA (ClinVar accession SCV001245412). An induced pluripotent stem cell (iPSC) line, ZZUNEUi010-A,^1^ which also harbors the c.1509dupA: p.R503fs mutation, was established in our previous study (Chen et al., 2020). The healthy control cell line, DYR0100, was provided by the Chinese Academy of Sciences Cell Bank.

### 2.2 Physicochemical properties and structure analysis

ProtParam was used for the analysis of physical and chemical properties.^2^ ProtScale was used for hydrophobic/hydrophilic analysis.^3^ NetPhos 2.0 was used for phosphorylation site analysis.^4^ NetOGlyc 4.0 Server^5^ and NetNGlyc 1.0 Server^6^ were used for O-glycosylation and N-glycosylation site analysis, respectively. SOPMA was used for secondary structure analysis of proteins.^7^ PSIpred was used for the tertiary structure analysis of proteins.^8^ Swiss-model^9^ was used for homologous modeling. Pymol was used for structural display and homologous comparison of advanced structures.

### 2.3 MNs generated from iPSC line ZZUNEUi010-A and DYR0100

iPSCs can be differentiated into MNs within approximately 28 ~ 30 days *in vitro* by following the protocol described by Du et al. (2015). This differentiation involves the addition of specific signaling molecules in a stepwise process when cells are cultured in six-well plates. Initially, from days 0 to 6, the basal medium (comprising DMEM/F12, neurobasal, N2, B27, ascorbic acid, β-mercaptoethanol, GlutaMAX, and P/S) is supplemented with 2 μL of SB431542, 3 μL of CHIR 99021, and 0.2 μL of LDN 193189 to induce the iPSCs into neural precursor cells (NPCs). At day 7, the medium is further supplemented with 2 μL of compound E, 2 μL of SB431542, 0.2 μL of LDN193189, 1 μL of SAG, 1 μL of CHIR 99021, and 1 μL of RA to progress NPCs into motor neural precursor cells (MNPCs). From day 13 to day 20, the medium is changed to include 1 μL of RA, 1 μL of SAG, and 0.1 μM compound E to maintain MNPCs. From day 21 to day 28, to induce MNPCs into MNs, the medium is adjusted to include 1 μL of RA, 1 μL of SAG, 0.1 μM compound E, 10 ng/mL of GDNF, 10 ng/mL of BDNF, and 10 ng/mL of CDNF. Post-day 28 ~ 30, MNs are ready for further identification and experimental use.

### 2.4 Motor neuron grouping and administration

The MNs derived from ZZUNEUi010-A and DYR0100 cell lines are referred to as CMF-MN and D01-MN, respectively. Three independent experiments were conducted to analyze these MNs through immunofluorescence staining and transcriptome sequencing, with four biological replicates for each group (CMF-MN and D01-MN). Neuroelectrophysiology experiments using the microelectrode array (MEA) system had eight biological replicates per group (CMF-MN, D01-MN, CMF-MN + T, and D01-MN + T), with the drug experiment groups (CMF-MN + T and D01-MN + T) exposed to 10 μM ropinirole. Calcium active imaging experiments were conducted with three biological replicates per group (CMF-MN and D01-MN).

### 2.5 Microelectrode array

Neuronal activity recordings were carried out using the Maestro MEA system (Axion Biosystems, Atlanta, GA, USA) with AxIS software. The induction of MNs was performed in 48-well MEA plates, coated with 0.15% polyethyleneimine and 50× Matrigel, seeding each well with 2.0 × 10^5^ cells and changing half of the medium every 3 days. MN cultures and MEA recordings were maintained at 37°C using the HopCell™ human MN medium (Cat. no. HopCell-MNM-500 Kit, Hopstem Biotechnology), following the manufacturer’s protocols. During experiments, spontaneous activity was recorded for 10 min, with a spike detection threshold set at 5.5 times the noise’s standard deviation. The CytoView MEA 48-well plate (Cat. no. M768-tMEA-48 W), suitable for the Maestro MEA system, features 16 electrodes per well. An active electrode is one that registers at least five spikes per minute on average. The number of spikes (number of active electrodes), weighted mean firing rate (spikes per second), number of bursts (synchrony index), and burst frequency (bursts per second) were analyzed using the Axion Integrated Studio program (Axion Biosystems).

### 2.6 Calcium activity imaging

On day 28 *in vitro* (MNs-DIV 0), MNPs were seeded in Matrigel (Corning #354230)-coated glass Petri dishes and cultured with HopCell® Human MN differentiation culture medium. The culture medium was subsequently replaced with the neurobasal medium. By DIV 30, we performed calcium signal recording and N-methyl-D-aspartate (NMDA) stimulation experiments. We removed the existing medium from the Petri dish and carefully added 300–500 μL of preheated (at 37°C) magnesium-free extracellular solution. Baseline recordings were made via video microscopy for a duration of 50–200 s. Without pausing the video, 300–500 μL of a mixture containing NMDA (200 μM; Sigma #M3262) and Glycine (20 μM) (Sigma#G7126) was added. An increase in neuronal discharge was observed and recorded for 150–200 s. After introducing 100 μL of MK801 (5 μM) (MCE#HY-15084-10) near the neuron, a gradual decrease in the intensity of neuronal discharge was noted and recorded for 150–200 s.

### 2.7 Statistical analysis

GraphPad Prism 6 statistical software was used for data analysis. Comparisons between two samples were made using a *t*-test, with a *p*-value of <0.05 deemed to indicate statistical significance.

## 3 Results

### 3.1 Physicochemical properties and structure of FUS^R503fs^

**Figure 1:**
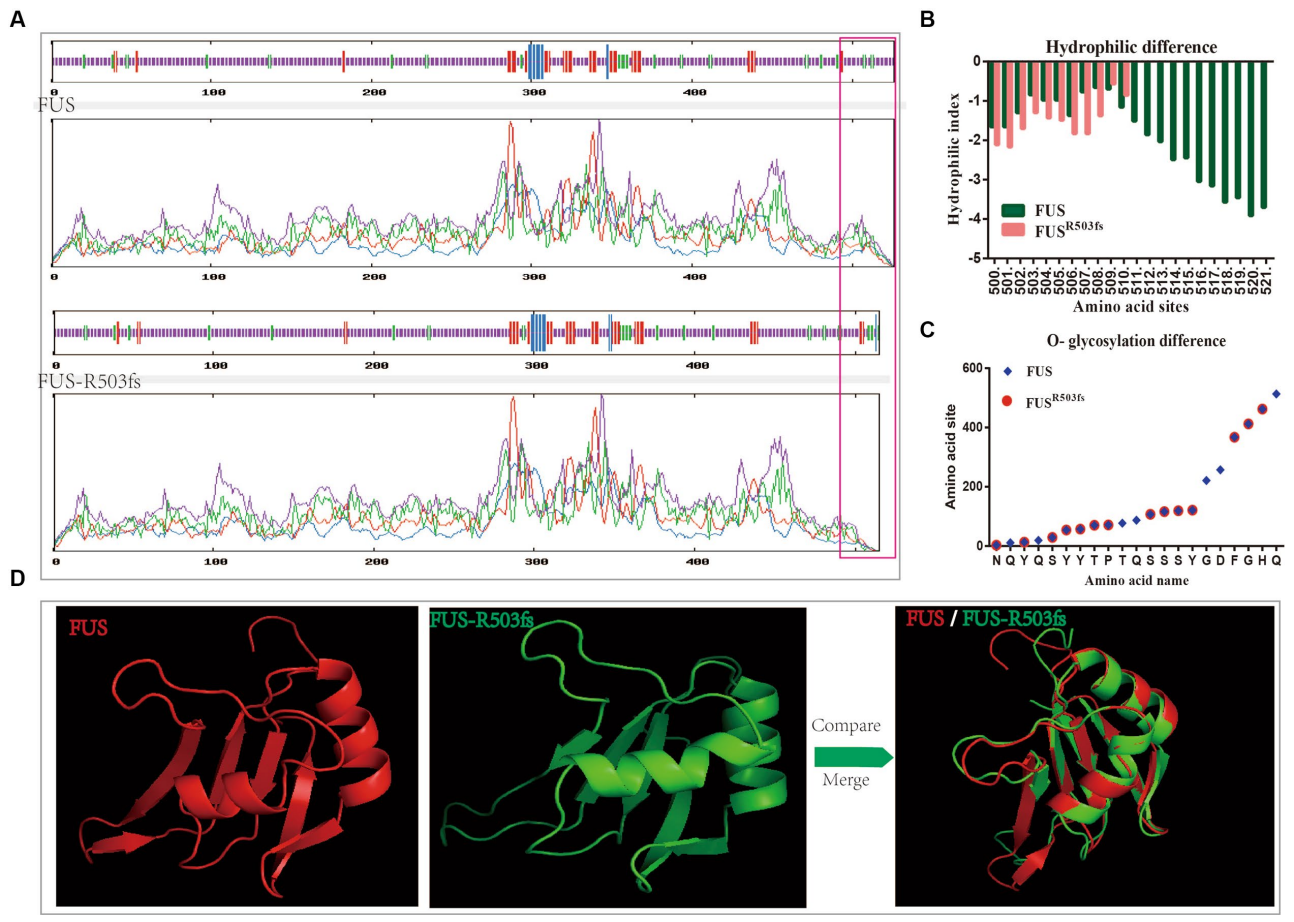
Analysis of physical structure and physicochemical properties of the FUS^R503fs^ protein. **(A)** The primary structure differences between FUS^R503fs^ and normal FUS mainly appeared at NLS, as shown in the red square. **(B)** Hydrophilic/hydrophobic chemical analysis revealed decreased hydrophilicity around and after the mutation site of the FUS^R503fs^ protein. **(C)** O-glycosylation sites of FUS^R503fs^ were significantly reduced compared to normal FUS. (**D**) 3D structures of FUS and FUS^R503fs^.

To further investigate the pathological mechanism of a novel gene mutation, we predicted the physical and chemical properties of mutant FUS using software. This novel mutation affected the secondary structure (Supplementary material 3) and physicochemical characteristics of FUS (Table 1). Hydrophobic/hydrophilic analysis revealed that both normal and mutant FUS proteins were hydrophilic, with reduced hydrophilicity at the NLS end of FUSR503fs compared to the normal FUS (Figure 1A). A marked decrease in hydrophilicity was observed around and beyond the mutation site of FUS (Figure 1B). The mutation primarily affected phosphorylation post-mutation site (Supplementary material 4). Glycosylation analysis indicated a significant reduction in O-glycosylation sites in FUS^R503fs^ compared with normal FUS (Figure 1C), while N-glycosylation remained unchanged (Supplementary material 6). The R503fs mutation in FUS altered the protein binding site, nucleic acid binding site, and disulfide bond formation site, suggesting an impact on the normal function of mutant FUS. The mutation’s effect on FUS’s secondary structure primarily occurred after the mutation site, resulting in tertiary structural differences not confined to the mutation’s vicinity. To provide a clearer visual representation, we reconstructed the three-dimensional structure of the mutant FUS for comparison with the normal FUS (Figure 1D).

**Table 1:** Physical and chemical properties of normal and mutant FUS.

Physical and chemical properties and characteristics	FUS	FUS^R503fs^
Number of amino acids	526	515
Molecular weight	53425.84	52312.66
Theoretical pI	9.4	9.32
Total number of negatively charged residues (Asp+Glu)	37	34
Total number of positively charged residues (Arg + Lys)	51	46
Estimated half-life	30 h	30 h
Total number of atoms	7,076	6,925
Instability index	57.11	58.44
Formula	C_2214_H_3314_N_736_O_800_S_1_	C_2180_H_3238_N_714_O_782_S_1_
Aliphatic index	13.38	14.43
Grand average of hydropathicity (GRAVY)	−1.319	−1.277
Phosphorylation sites of amino acids	86	84
O-Glycosylation sites of amino acids	21	14
Secondary structure of proteins		
Alpha helix (Hh)	2.28%	2.52%
Extended strand (Ee)	8.94%	9.32%
Beta turn (Tt)	7.41%	7.77%
Random coil (Cc)	81.37%	80.39%

### 3.2 Differentiation of motor neurons from iPSCs and identification of their maturity

**Figure 2:**
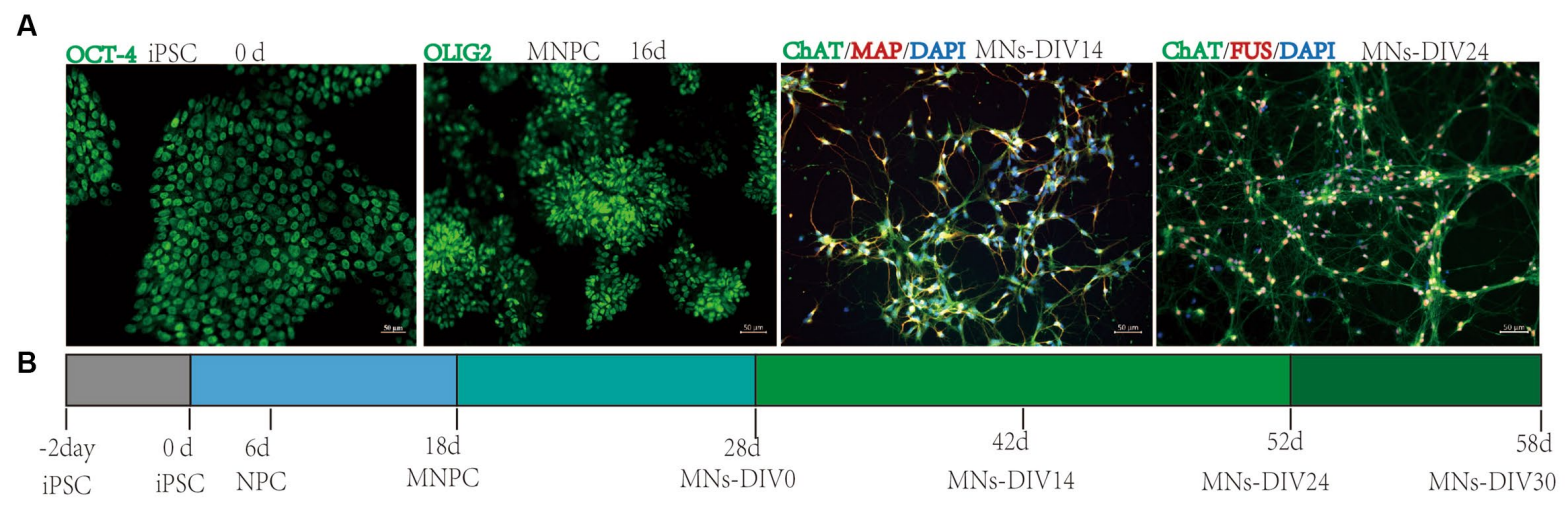
Motor neuron differentiation and experimental procedures. **(A)** Differentiation from iPSC into MNs. **(B)** Timeline of motor neuron differentiation and experimental procedures.

Fourteen days post-differentiation into MNs (MNs-DIV 14), the neurons were stained with cytoplasmic, cell membrane, and nucleus-specific MN markers by immunofluorescence (Supplementary material 7). This confirmed that the MNs were mature and highly expressed specific markers (Figure 2A). Our previous study observed abnormal FUS accumulation in FUS^R503fs^ MNs, with an increase in mRNA and protein expression (Chen et al., 2020). The timeline of motor neuron differentiation and experimental procedures are shown in Figure 2B. The MEA experiment started from MNs-DIV24 and calcium activity imaging was performed at MNs-DIV30.

### 3.3 MEA showed that the electrophysiological activity of motor neurons with FUS^R503fs^ mutation was decreased

**Figure 3:**
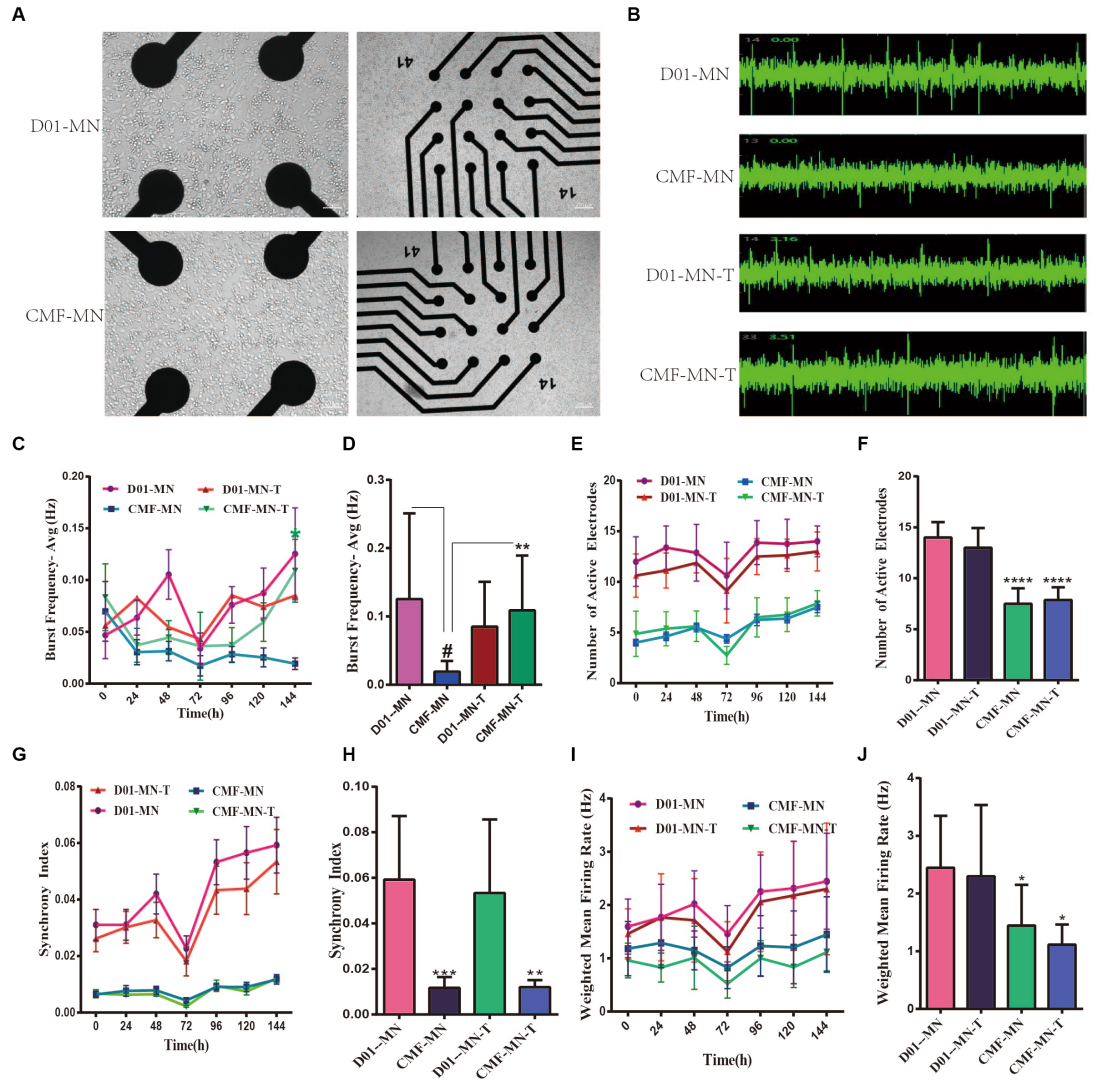
MEA shows the electrical activity of FUS^R503fs^ mutant neurons decreased and partially reversed by ropinirole. **(A)** Bright-field picture of motor neurons on MEA. **(B)** Sample images of motor neuron spontaneous electrical activity. **(C,D)** The burst frequency decreased significantly (**p < 0.01); Ropinirole reversed the decrease in burst frequency in FUS^R503fs^ motor neurons (#*p* < 0.01). **(E,F)** The number of active electrodes in FUS^R503fs^ motor neurons was significantly lower than that of normal motor neurons (****p < 0.0001); **(G,H)** The synchrony index of FUS^R503fs^ neurons was decreased significantly (***p < 0.001); **(I,J)** The weighted mean firing rate of FUS^R503fs^ neurons was decreased significantly (*p < 0.05).

We assessed the electrical activity of MN networks using the MEA system (Figure 3A). We detected action potentials (“spikes”) and groups of action potentials (“bursts”) (Figure 3B) in MN networks derived from two cell lines (CMF-MNs and D01-MNs) over several days *in vitro* (DIV 24, DIV25, DIV26, DIV27, DIV28, DIV29, and DIV30). We calculated the mean firing rate (Hz) as the ratio of the total number of spikes recorded to the duration of the recording. We measured four classical observation indices with the Axion Integrated Studio program (Axion Biosystems): the number of spikes (number of active electrodes), weighted mean firing rate (spikes per second), number of bursts (synchrony index), and burst frequency (bursts per second). The results showed that the indices for FUS^R503fs^ mutant neurons were significantly lower than those for the control group (Figures 3C–J).

### 3.4 Calcium activity imaging revealed a reduced frequency of calcium signaling activity in FUS^R503fs^-mutated MNs

**Figure 4:**
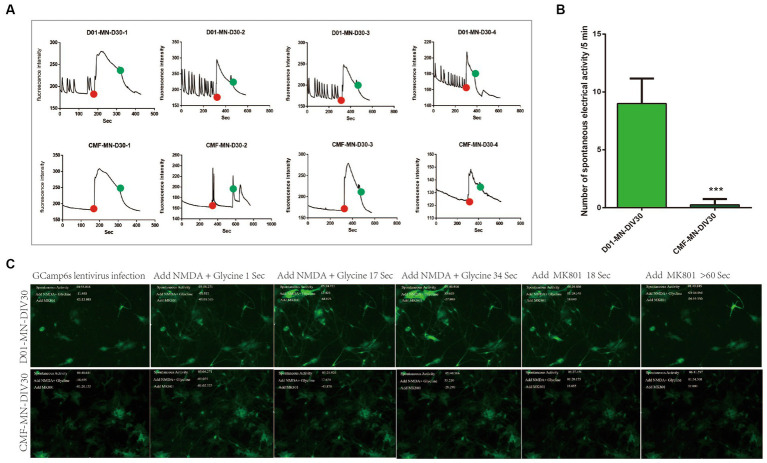
Calcium activity imaging results. **(A,B)** The spontaneous electrical signal of FUS^R503fs^ motor neurons was significantly reduced (***p < 0.001). The red dot indicates the time point of agonist administration (glutamic acid + glycine), and the green dot indicates the time point of inhibitor administration (MK801). **(C)** Example photographs of the evolution of calcium activity signals in two groups of motor neurons.

To further explore the effect of the FUS^R503fs^ mutation on neural electrical activity, we performed calcium activity imaging on mature cultured neurons (DIV 30). We observed that the spontaneous calcium signal in FUS^R503fs^ mutant neurons was significantly diminished. After sequential administration of the NMDAR agonist glutamic acid + glycine and the NMDAR inhibitor MK801, no differences in calcium activity were noted between the two groups. This indicates no significant abnormalities in the glutamate receptor system, suggesting that the FUS^R503fs^ mutation’s effect on neural calcium activity might be due to other factors (Figure 4).

### 3.5 Ropinirole treatment alleviated the electrophysiological decline in FUS^R503fs^ neurons to a certain extent

**Figure 5:**
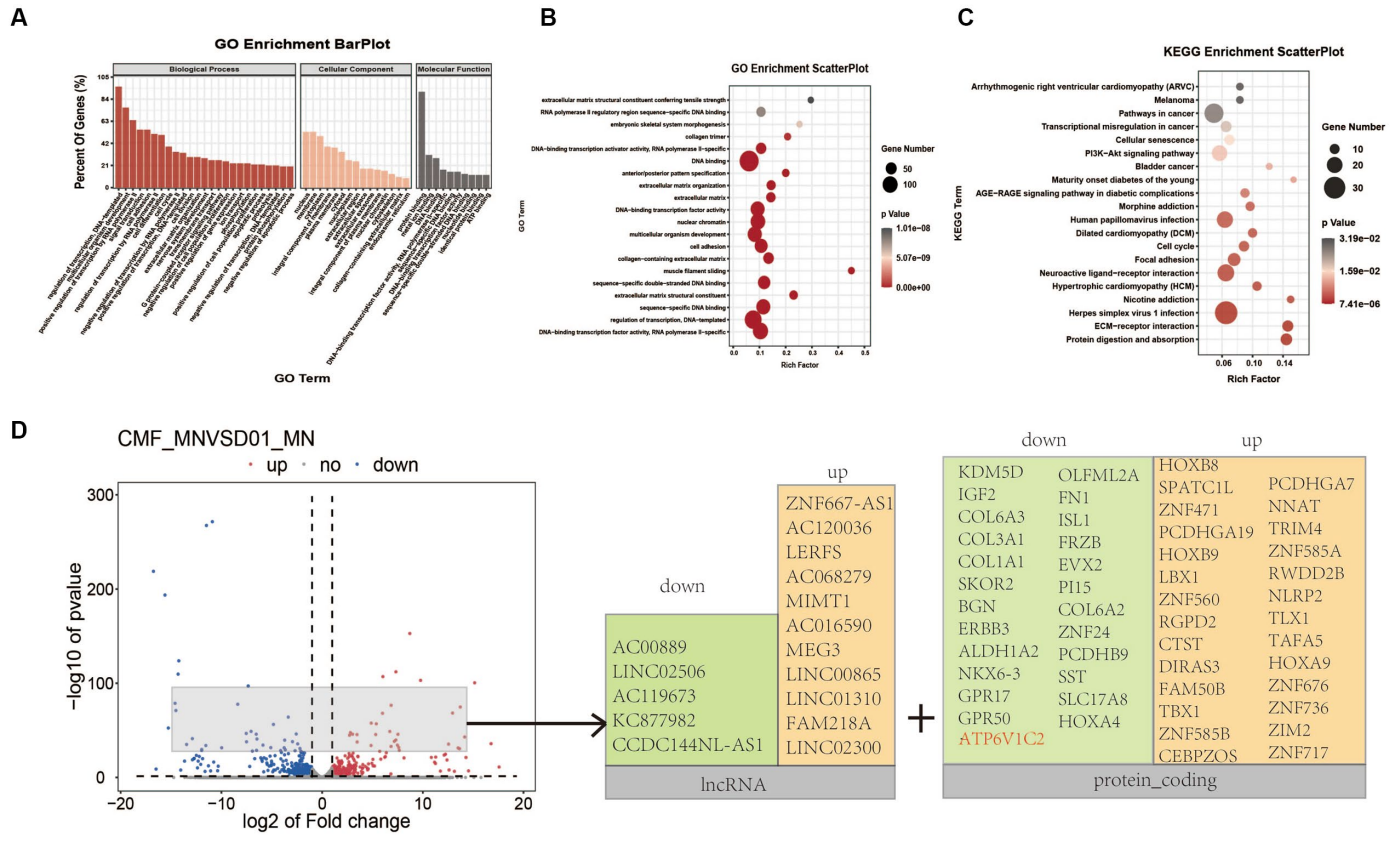
Transcriptomic analysis of FUS^R503fs^-mutated motor neurons and normal control group. **(A)** The analysis of cell components suggested that differentially expressed genes were mainly involved in DNA transcription regulation and protein binding. **(B)** The GO enrichment analysis suggested that differentially expressed genes were mainly concentrated in DNA/RNA binding and regulation. **(C)** The KEGG enrichment analysis suggested that differentially expressed genes mainly affect receptor and ligand interaction pathways of neural activation and viral infection pathways. **(D)** The volcano plot showed that the top significantly upregulated or downregulated genes were mainly lncRNA and protein-coding genes after removing sex-related and pseudogenes.

Previous studies have demonstrated that iPSC-induced MNs can serve as a tool for drug screening in ALS treatment, with ropinirole showing potential pharmacological value (Fujimori et al., 2018). In our experiment, from MNs-DIV25, we added 10 μM ropinirole to the MEA test group’s drug experimental group for 144 h. The results indicated that ropinirole mitigated the decline in burst frequency observed in the mutant group, with no significant differences in other indices such as number of electrodes, synchrony index, or weighted mean firing rate (Figure 5).

### 3.6 Transcriptome analysis suggested that FUS^R503fs^ mainly caused the downregulation of neural function-related genes and the upregulation of stress-related genes

Transcriptome sequencing of mature cultured neurons (DIV 25) revealed that 610 genes were downregulated and 453 genes were upregulated in the FUS^R503fs^ mutant group (Figure 5). The differentially expressed genes were primarily involved in transcriptional regulation, DNA templating, nuclear function, and protein binding (Figures 5A,B). Genes related to neural function were significantly downregulated, whereas genes associated with viral infection and stress pathways were upregulated (Figure 5C). The top significantly upregulated or downregulated genes were mainly lncRNA and protein-coding genes after removing sex-related genes and pseudogenes (Figure 5D). For example, ATP6V1C2, a gene linked to autophagolysosome, receptor and ligand interactions, vesicular transport, and energy metabolism, was significantly reduced (Log2fc < −2, *p* < 0.0001).

## 4 Discussion

The normal full-length FUS protein consists of 256 amino acid residues, including an LC domain, three RGG domains (RGG1, RGG2, and RGG3), an RRM domain, a zinc finger (ZnF) domain, and a NLS. The RGG1-RRM domain can bind RNA, while the RGG2-ZnF-RGG3 domain has a strong affinity for RNA and single-stranded DNA (ssDNA) (Schwartz et al., 2015). The majority of ALS-associated FUS mutations are missense mutations affecting the NLS. Additionally, some frameshift mutations resulting in complete NLS deletions have been reported. Our study identified a novel mutation, c.1509dupA:pR503fs, located in the NLS, leading to a truncated FUS protein of 503 amino acids with an aberrant sequence of RWLWPWQDGFQG. The addition of a novel “tail” of amino acids at the FUS C-terminus due to frameshift would alter the properties of truncated FUS proteins. Evidence suggests that mutations such as c.1432_1478del, c.1506dupA, and c.1509_1510delAG result in a frameshift peptide WLWPWQDGFQG, which may increase nuclear retention of mutant FUS (An et al., 2020). The c.1509dupA mutation results in truncated FUS proteins with a “tail” RWLWPWQDGFQG containing the peptide WLWPWQDGFQG, possibly explaining the accumulation of FUS in the nucleus and cytoplasm of differentiated MNs in our previous study (Chen et al., 2020). Although the presence of this peptide can partially rescue cytoplasmic FUS deposition, the accumulation of mutant FUS in the nucleus can lead to toxic gain-of-function effects. FUS nuclear import is mediated by its NLS sequence binding to the nuclear transporter TNPO1 (Transportin-1 and TNPO1) (Springhower et al., 2020), while nuclear export is independent of Exportin 1 (Exportin 1 and XPO1) (Fahrenkrog and Harel, 2018). This disruption severely affects the normal shuttling of FUS between the cytoplasm and nucleus, impacting its physiological function and causing cytotoxicity. The severity and rapid progression of ALS associated with this mutation may result from a combination of loss-of-function and gain-of-toxic function.

Changes in physicochemical properties and modification sites likely contribute to the pathophysiological effects of FUS mutations. Phosphorylation of FUS can occur in specific domains to prevent oligomerization, which affects its ability to regulate transcription. Arginine residues in the LC domain are also necessary for FUS maturation in cellular SGs (Bogaert et al., 2018). FUS functions not only through chemical interactions but also through its unique physical structure. The ability of FUS to bind G-quadruplexes depends on the β-spiral structure of the RGG domain (Yagi et al., 2018). Specifically, modified tyrosine in the RGG domain of FUS binds to G-quadruplex RNA, recognizing the 2′-OH of the ribose in the loops of G-quadruplexes (Takahama and Oyoshi, 2013). Our physicochemical and structural analyses (Figure 1) revealed significant differences in FUS^R503fs^, particularly in the tertiary structure, hydrophilicity/hydrophobicity, and O-glycosylation sites. Previous studies have shown that EOGT mutations in ALS reduce O-GlcNAcylation, leading to the hyperphosphorylation of neurofilaments (Moll et al., 2019). Additionally, de-glycosylation is associated with a block in endoplasmic reticulum-to-Golgi traffic during autophagy progress (Fahie and Zachara, 2016). The structural abnormalities, reduced phosphorylation sites, and decreased hydrophilicity observed in the NLS domain of FUS^R503fs^ could be related to nuclear import disorders and dysregulation of transcriptional regulation. The significant reductions in O-glycosylation sites and hydrophilic changes may contribute to the aggregation of aberrant proteins and FUS.

Our MEA and calcium activity imaging experiments on MNs showed a significant decrease in the spontaneous electrical activity and spontaneous calcium activity signal of FUS^R503fs^ mutant neurons (Figure 3). The decrease in MN spontaneous electrical activity (DIV 3–4 weeks) occurred earlier than that of iPSCs derived from FUS^R521L,R521C,R495QfsX527^ genetic mutations, which typically occurs between DIV 7 and 10 weeks (Gunes et al., 2020). Considering that ALS patients usually develop symptoms in adolescence or adulthood and that iPSC-induced neurons can survive and grow *in vitro* for more than 70 days, we have performed MEA since DIV 24 and calcium activity imaging since DIV 30 to observe more obvious pathological manifestations. In our experiment, FUS^R503fs^ MNs (>DIV 24) exhibited hypoexcitability, while it has been reported that iPSC-derived MN genome-edited from a healthy cell line (FUS^H517D/H517D^) showed hyperexcitability on day 7 by MEA and increased calcium activity on day 14 by calcium activity imaging (Kondo et al., 2023). The discrepancy in results may be due to the different lengths of culture time *in vitro*, suggesting that the excitability and activity of neurons in the early and late stages of the disease may differ. This hypothesis needs to be confirmed by large-scale studies with time gradients.

Transcriptomic analysis (Figure 4) of MNs revealed upregulated genes associated with viral infection and a downregulation of genes related to the maintenance of neural function, such as the ATP6V1C2 gene. ATP6V1C2 (ATPase H^+^ transporting V1 subunit C2) belongs to the vacuolar ATPase (v-ATPase) family and is significantly reduced in FUS^R503fs^ neurons mutated with FUS^R503fs^ (Log2fc < −2, *p* < 0.0001). Simultaneously, the expression of autophagosome/lysosome network components, ATP-binding cassette subfamily A8(ABCA8) and ATP-binding cassette subfamily C6(ABCC6) also declined (Log2fc < −1, *p* < 0.01). Maintaining a lysosomal acidic environment and autophagy flow is an energy-intensive process, relying on ATP breakdown facilitated by ABCA8 and ABCC6 (França et al., 2020). A study in Nature Cell Biology on the autophagy phenotype related to a common ALS-associated mutation in UBQLN2 highlights the role of abnormal v-ATPase-MTOR-mediated autophagy in ALS pathogenesis (Yang and Klionsky, 2020). Ubiquilins are crucial for v-ATPase-mediated lysosomal acidification (Senturk et al., 2019). A genome-wide CRISPR screen identified v-ATPase as a potential drug target to reduce levels of the ALS protein ataxin-2 (Kim et al., 2022), suggesting that FUS^R503fs^ mutations likely affect the autophagic lysosomal system.

We evaluated the therapeutic impact of ropinirole on induced MNs using MEA analysis. The results indicated that the decrease in burst frequency observed in mutated neurons could be partially reversed (Figure 5). Because only one concentration and one treatment duration were tested, these results might not fully represent the drug’s efficacy. However, they suggest that this cell line could serve as a tool for drug screening and hint at the potential therapeutic value of ropinirole in FUS-ALS. Ropinirole, a non-ergot dopamine receptor agonist, has been reported to activate autophagy, possibly through a Beclin-1-dependent pathway (Okano et al., 2020). It would be interesting to explore if ropinirole-induced activation of D2R/D3R receptors could induce autophagy, leading to the degradation of abnormal RNA–protein complexes in ALS MNs.

## Data availability statement

The data presented in this study has been deposited in the SRA on NCBI repository, accession number PRJNA1097276.

## Ethics statement

The studies involving humans were approved by Ethics Committee of Shanghai Sixth People’s Hospital. The studies were conducted in accordance with the local legislation and institutional requirements. The participants provided their written informed consent to participate in this study.

## Author contributions

LC: Data curation, Formal analysis, Funding acquisition, Writing – original draft, Writing – review & editing. GC: Methodology, Software, Writing – review & editing. MZ: Methodology, Writing – review & editing. XZ: Supervision, Writing – review & editing.

## Footnotes

^1^ https://hpscreg.eu/cell-line/ZZUNEUi010-A

^2^ https://web.expasy.org/protparam/

^3^ https://web.expasy.org/protscale/

^4^ http://www.cbs.dtu.dk/services/NetPhos/

^5^ http://www.cbs.dtu.dk/services/NetOGlyc/

^6^ http://www.cbs.dtu.dk/services/NetNGlyc/

^7^ https://npsa-prabi.ibcp.fr/cgi-bin/npsa_automat.pl?page=npsa_sopma.html

^8^ http://bioinf.cs.ucl.ac.uk/psipred/psiform.html

^9^ https://www.baidu.com/link?url=a-tuifFkzGDy8dBuJYYMGHkb7P_piysqDB3RhoeuMs2RniJP22tC8J4tiQ7aWgOF&wd=&eqid=fff5783c000451f1000000045e9d06c4
