# Supplementary material for: Modeling sporadic juvenile ALS in iPSC-derived motor neurons explores the pathogenesis of FUSR503fs mutation
Source: Front Cell Neurosci. 2024 Apr 22;18:1364164. doi: 10.3389/fncel.2024.1364164 (PMC11070534; doi:10.3389/fncel.2024.1364164)
Supplement: Supplementary file 1 [file Data_Sheet_1.doc]

**Sup.1 DNA sequence**

**DNA of Normal *FUS*:**

ATGGCCTCAAACGATTATACCCAACAAGCAACCCAAAGCTATGGGGCCTACCCCACCCAGCCCGGGCAGGGCTATTCCCAGCAGAGCAGTCAGCCCTACGGACAGCAGAGTTACAGTGGTTATAGCCAGTCCACGGACACTTCAGGCTATGGCCAGAGCAGCTATTCTTCTTATGGCCAGAGCCAGAACACAGGCTATGGAACTCAGTCAACTCCCCAGGGATATGGCTCGACTGGCGGCTATGGCAGTAGCCAGAGCTCCCAATCGTCTTACGGGCAGCAGTCCTCCTACCCTGGCTATGGCCAGCAGCCAGCTCCCAGCAGCACCTCGGGAAGTTACGGTAGCAGTTCTCAGAGCAGCAGCTATGGGCAGCCCCAGAGTGGGAGCTACAGCCAGCAGCCTAGCTATGGTGGACAGCAGCAAAGCTATGGACAGCAGCAAAGCTATAATCCCCCTCAGGGCTATGGACAGCAGAACCAGTACAACAGCAGCAGTGGTGGTGGAGGTGGAGGTGGAGGTGGAGGTAACTATGGCCAAGATCAATCCTCCATGAGTAGTGGTGGTGGCAGTGGTGGCGGTTATGGCAATCAAGACCAGAGTGGTGGAGGTGGCAGCGGTGGCTATGGACAGCAGGACCGTGGAGGCCGCGGCAGGGGTGGCAGTGGTGGCGGCGGCGGCGGCGGCGGTGGTGGTTACAACCGCAGCAGTGGTGGCTATGAACCCAGAGGTCGTGGAGGTGGCCGTGGAGGCAGAGGTGGCATGGGCGGAAGTGACCGTGGTGGCTTCAATAAATTTGGTGGCCCTCGGGACCAAGGATCACGTCATGACTCCGAACAGGATAATTCAGACAACAACACCATCTTTGTGCAAGGCCTGGGTGAGAATGTTACAATTGAGTCTGTGGCTGATTACTTCAAGCAGATTGGTATTATTAAGACAAACAAGAAAACGGGACAGCCCATGATTAATTTGTACACAGACAGGGAAACTGGCAAGCTGAAGGGAGAGGCAACGGTCTCTTTTGATGACCCACCTTCAGCTAAAGCAGCTATTGACTGGTTTGATGGTAAAGAATTCTCCGGAAATCCTATCAAGGTCTCATTTGCTACTCGCCGGGCAGACTTTAATCGGGGTGGTGGCAATGGTCGTGGAGGCCGAGGGCGAGGAGGACCCATGGGCCGTGGAGGCTATGGAGGTGGTGGCAGTGGTGGTGGTGGCCGAGGAGGATTTCCCAGTGGAGGTGGTGGCGGTGGAGGACAGCAGCGAGCTGGTGACTGGAAGTGTCCTAATCCCACCTGTGAGAATATGAACTTCTCTTGGAGGAATGAATGCAACCAGTGTAAGGCCCCTAAACCAGATGGCCCAGGAGGGGGACCAGGTGGCTCTCACATGGGGGGTAACTACGGGGATGATCGTCGTGGTGGCAGAGGAGGCTATGATCGAGGCGGCTACCGGGGCCGCGGCGGGGACCGTGGAGGCTTCCGAGGGGGCCGGGGTGGTGGGGACAGAGGTGGCTTTGGCCCTGGCAAGATGGATTCCAGGGGTGAGCACAGACAGGATCGCAGGGAGAGGCCGTATTAA

**DNA of mutant *FUS* (c.1509dupA)**：

ATGGCCTCAAACGATTATACCCAACAAGCAACCCAAAGCTATGGGGCCTACCCCACCCAGCCCGGGCAGGGCTATTCCCAGCAGAGCAGTCAGCCCTACGGACAGCAGAGTTACAGTGGTTATAGCCAGTCCACGGACACTTCAGGCTATGGCCAGAGCAGCTATTCTTCTTATGGCCAGAGCCAGAACACAGGCTATGGAACTCAGTCAACTCCCCAGGGATATGGCTCGACTGGCGGCTATGGCAGTAGCCAGAGCTCCCAATCGTCTTACGGGCAGCAGTCCTCCTACCCTGGCTATGGCCAGCAGCCAGCTCCCAGCAGCACCTCGGGAAGTTACGGTAGCAGTTCTCAGAGCAGCAGCTATGGGCAGCCCCAGAGTGGGAGCTACAGCCAGCAGCCTAGCTATGGTGGACAGCAGCAAAGCTATGGACAGCAGCAAAGCTATAATCCCCCTCAGGGCTATGGACAGCAGAACCAGTACAACAGCAGCAGTGGTGGTGGAGGTGGAGGTGGAGGTGGAGGTAACTATGGCCAAGATCAATCCTCCATGAGTAGTGGTGGTGGCAGTGGTGGCGGTTATGGCAATCAAGACCAGAGTGGTGGAGGTGGCAGCGGTGGCTATGGACAGCAGGACCGTGGAGGCCGCGGCAGGGGTGGCAGTGGTGGCGGCGGCGGCGGCGGCGGTGGTGGTTACAACCGCAGCAGTGGTGGCTATGAACCCAGAGGTCGTGGAGGTGGCCGTGGAGGCAGAGGTGGCATGGGCGGAAGTGACCGTGGTGGCTTCAATAAATTTGGTGGCCCTCGGGACCAAGGATCACGTCATGACTCCGAACAGGATAATTCAGACAACAACACCATCTTTGTGCAAGGCCTGGGTGAGAATGTTACAATTGAGTCTGTGGCTGATTACTTCAAGCAGATTGGTATTATTAAGACAAACAAGAAAACGGGACAGCCCATGATTAATTTGTACACAGACAGGGAAACTGGCAAGCTGAAGGGAGAGGCAACGGTCTCTTTTGATGACCCACCTTCAGCTAAAGCAGCTATTGACTGGTTTGATGGTAAAGAATTCTCCGGAAATCCTATCAAGGTCTCATTTGCTACTCGCCGGGCAGACTTTAATCGGGGTGGTGGCAATGGTCGTGGAGGCCGAGGGCGAGGAGGACCCATGGGCCGTGGAGGCTATGGAGGTGGTGGCAGTGGTGGTGGTGGCCGAGGAGGATTTCCCAGTGGAGGTGGTGGCGGTGGAGGACAGCAGCGAGCTGGTGACTGGAAGTGTCCTAATCCCACCTGTGAGAATATGAACTTCTCTTGGAGGAATGAATGCAACCAGTGTAAGGCCCCTAAACCAGATGGCCCAGGAGGGGGACCAGGTGGCTCTCACATGGGGGGTAACTACGGGGATGATCGTCGTGGTGGCAGAGGAGGCTATGATCGAGGCGGCTACCGGGGCCGCGGCGGGGACCGTGGAGGCTTCCGAGGGGGCCGGGGTGGTGGGGACAGAAGGTGGCTTTGGCCCTGGCAAGATGGATTCCAGGGGTGAGCACAGACAGGATCGCAGGGAGAGGCCGTATTAA

**Sup.2 Amino acid sequence**

**Normal FUS：**

MASNDYTQQATQSYGAYPTQPGQGYSQQSSQPYGQQSYSGYSQSTDTSGYGQSSYSSYGQSQNTGYGTQSTPQGYGSTGGYGSSQSSQSSYGQQSSYPGYGQQPAPSSTSGSYGSSSQSSSYGQPQSGSYSQQPSYGGQQQSYGQQQSYNPPQGYGQQNQYNSSSGGGGGGGGGGNYGQDQSSMSSGGGSGGGYGNQDQSGGGGSGGYGQQDRGGRGRGGSGGGGGGGGGGYNRSSGGYEPRGRGGGRGGRGGMGGSDRGGFNKFGGPRDQGSRHDSEQDNSDNNTIFVQGLGENVTIESVADYFKQIGIIKTNKKTGQPMINLYTDRETGKLKGEATVSFDDPPSAKAAIDWFDGKEFSGNPIKVSFATRRADFNRGGGNGRGGRGRGGPMGRGGYGGGGSGGGGRGGFPSGGGGGGGQQRAGDWKCPNPTCENMNFSWRNECNQCKAPKPDGPGGGPGGSHMGGNYGDDRRGGRGGYDRGGYRGRGGDRGGFRGGRGGGDRGGFGPGKMDSRGEHRQDRRERPY

**mutant FUS (R503fs)：**

MASNDYTQQATQSYGAYPTQPGQGYSQQSSQPYGQQSYSGYSQSTDTSGYGQSSYSSYGQSQNTGYGTQSTPQGYGSTGGYGSSQSSQSSYGQQSSYPGYGQQPAPSSTSGSYGSSSQSSSYGQPQSGSYSQQPSYGGQQQSYGQQQSYNPPQGYGQQNQYNSSSGGGGGGGGGGNYGQDQSSMSSGGGSGGGYGNQDQSGGGGSGGYGQQDRGGRGRGGSGGGGGGGGGGYNRSSGGYEPRGRGGGRGGRGGMGGSDRGGFNKFGGPRDQGSRHDSEQDNSDNNTIFVQGLGENVTIESVADYFKQIGIIKTNKKTGQPMINLYTDRETGKLKGEATVSFDDPPSAKAAIDWFDGKEFSGNPIKVSFATRRADFNRGGGNGRGGRGRGGPMGRGGYGGGGSGGGGRGGFPSGGGGGGGQQRAGDWKCPNPTCENMNFSWRNECNQCKAPKPDGPGGGPGGSHMGGNYGDDRRGGRGGYDRGGYRGRGGDRGGFRGGRGGGDRRWLWPWQDGFQG

**Sup.3** **Secondary structure (analysis by PSIPRED)**

**Normal FUS：There are 5 folds (yellow), 3 spirals (red), and the rest are curled.**


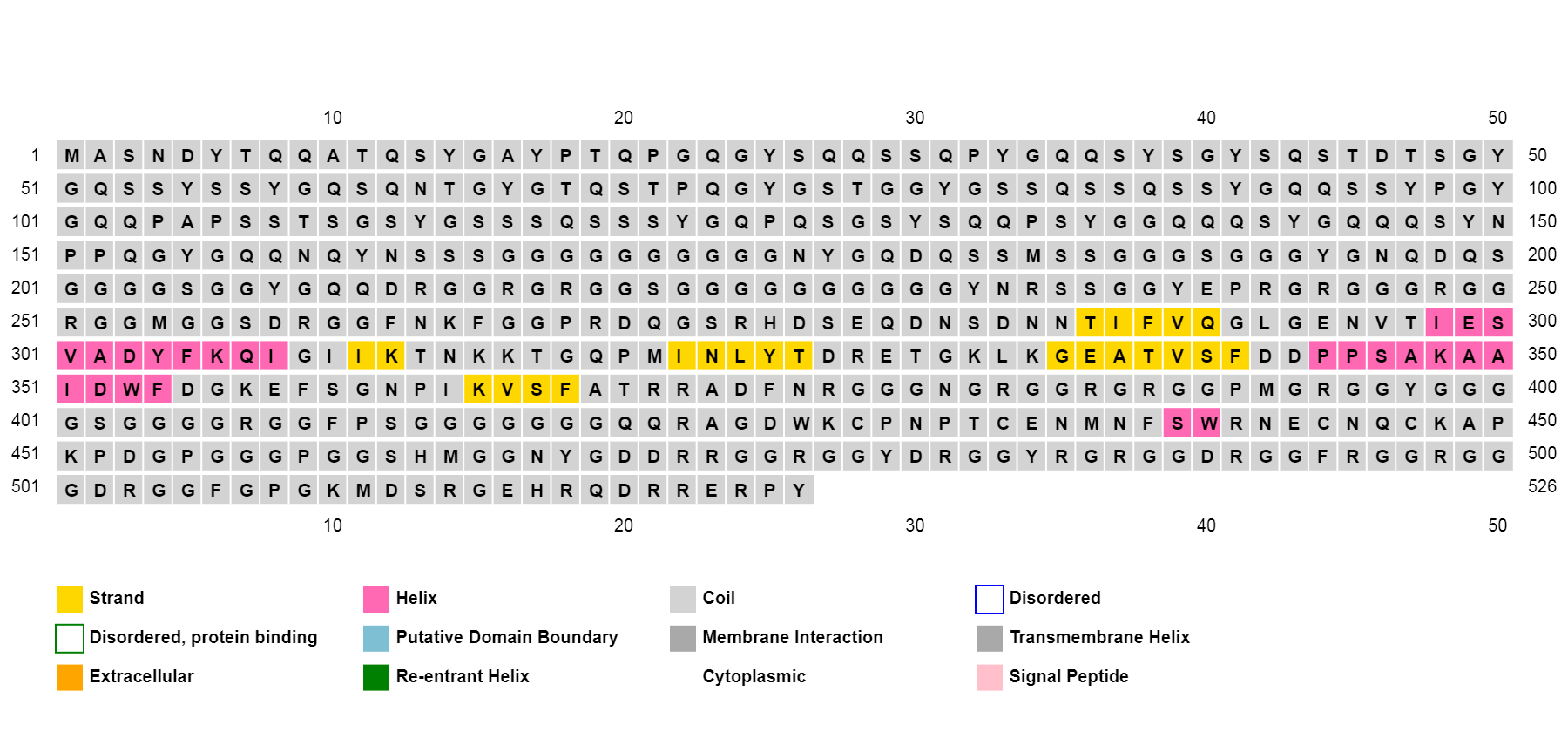


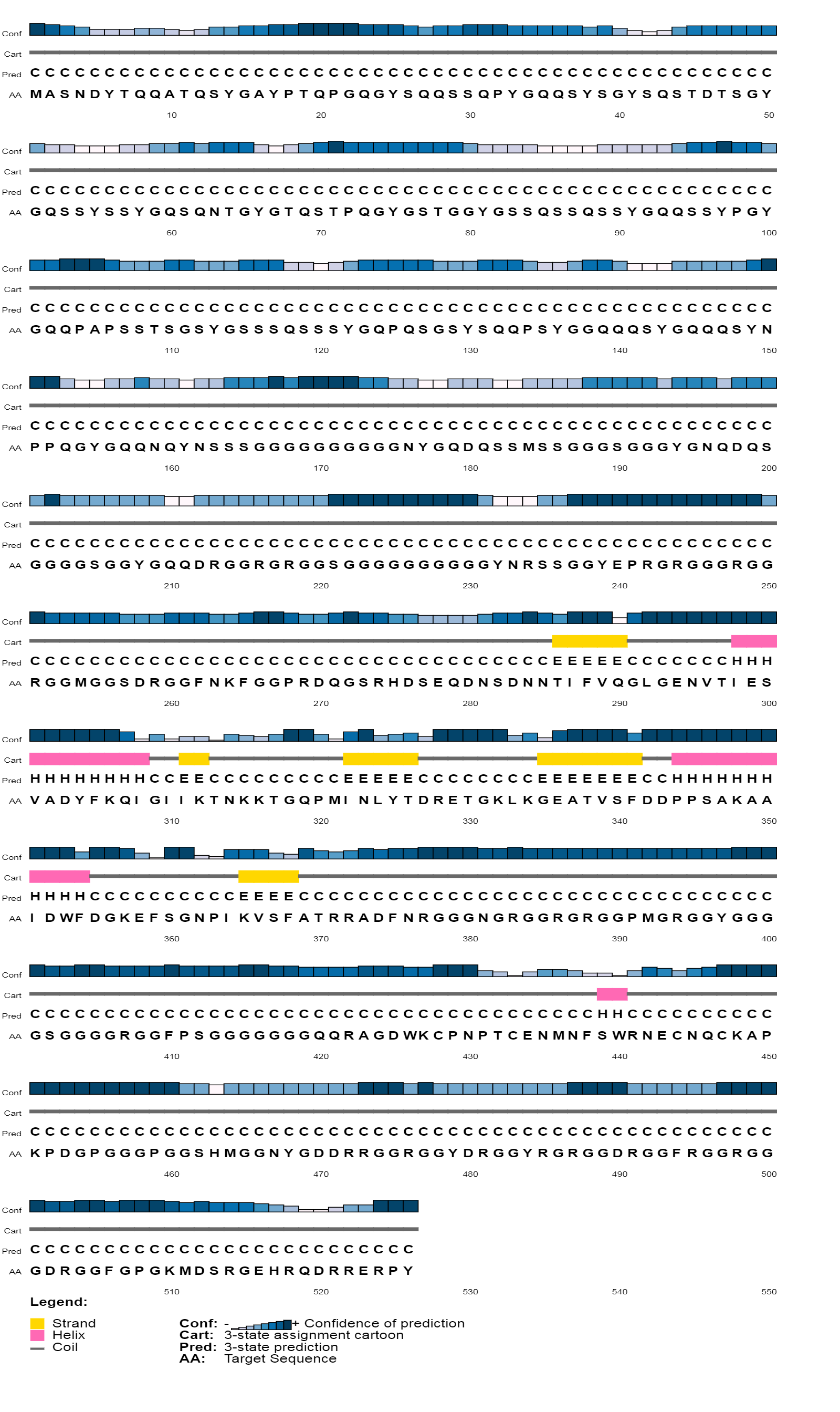


**FUS^R503fs^**

**There are 6 folds (yellow), 3 spirals (red), and the rest are curled.**


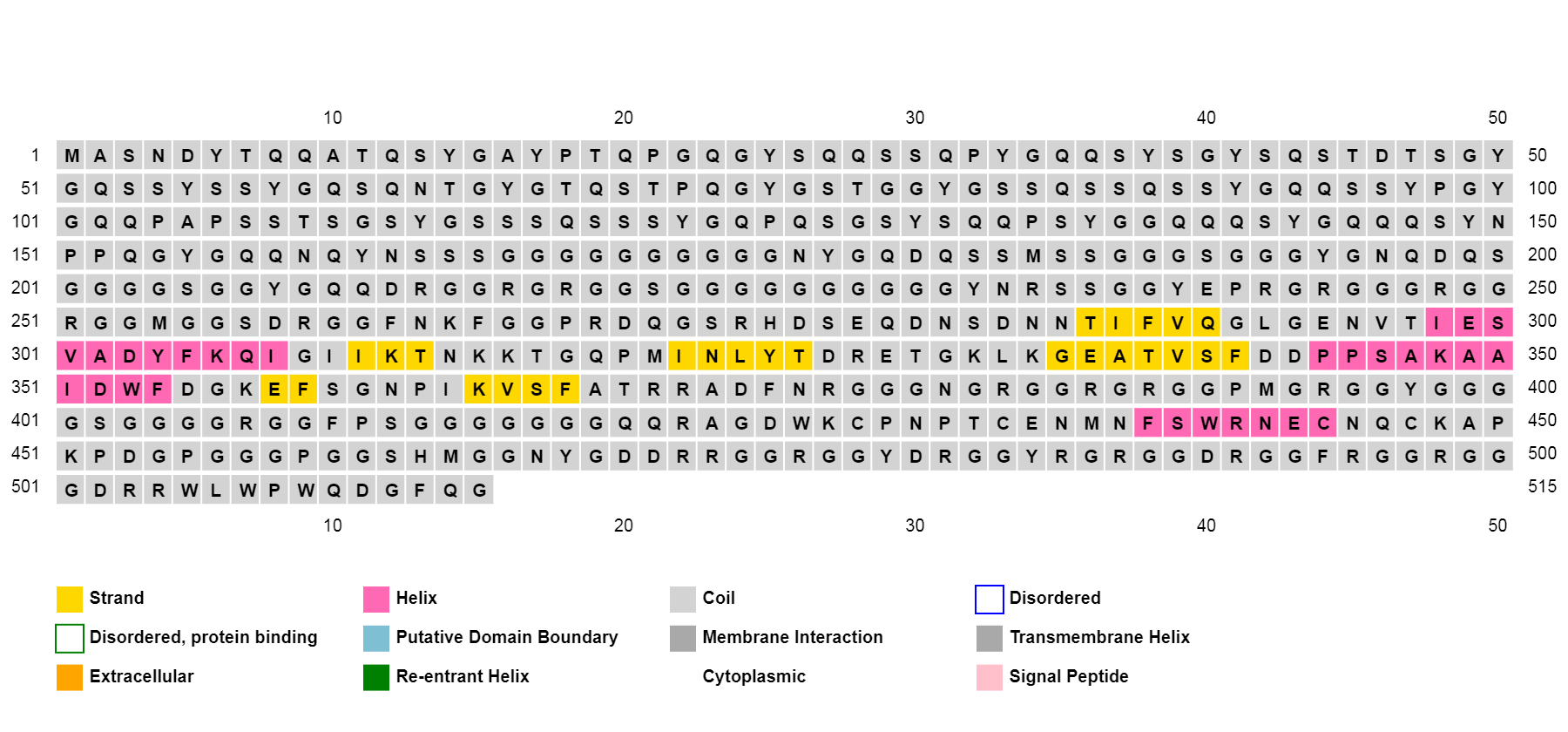


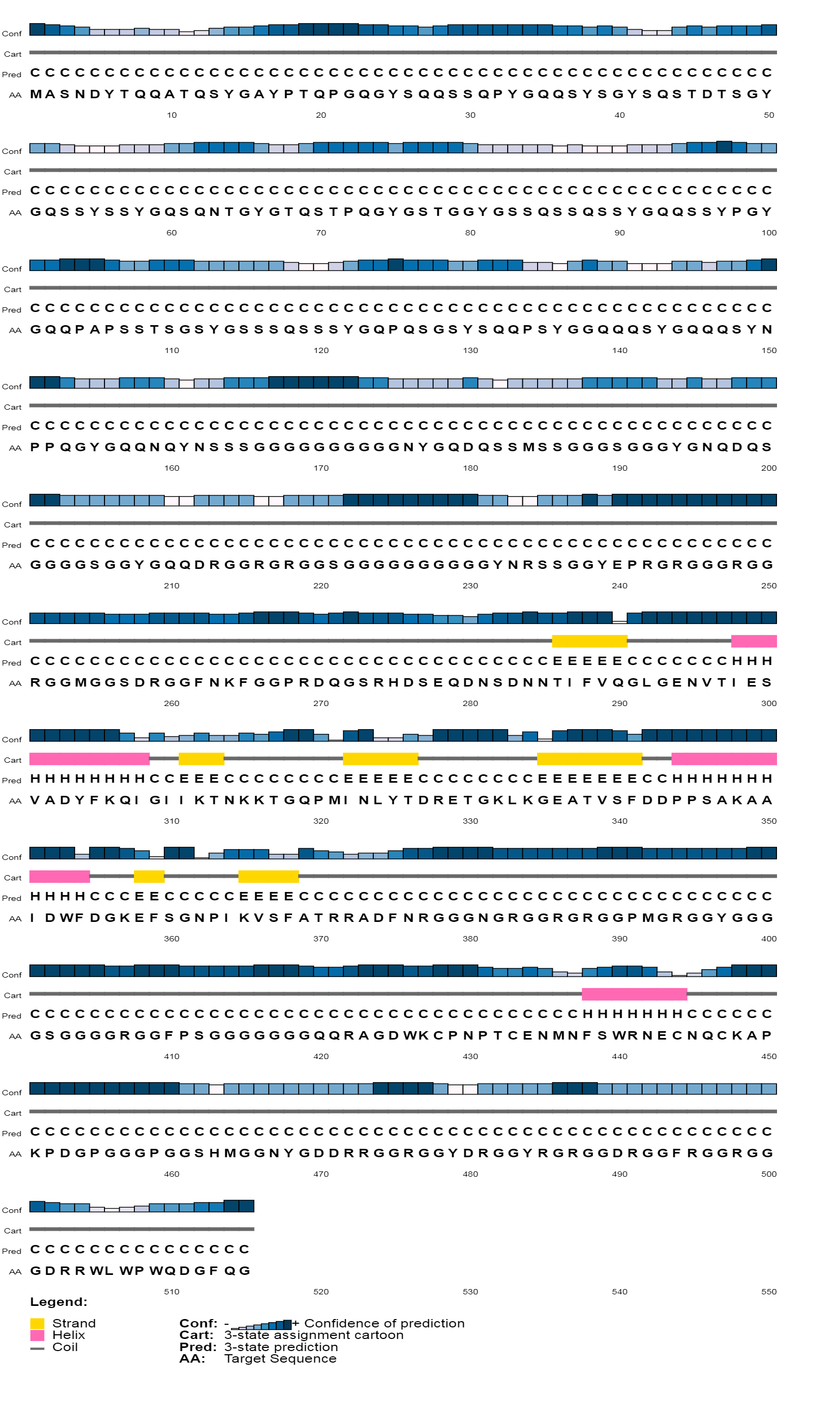


**Sup.4 Phosphorylation site analysis**

There was no significant change in phosphorylation sites before insertion mutation, but two phosphorylation sites were missing after insertion mutation.

**FUS**：86 phosphorylation sites

%1 .....YT...T.S.....T......S...S......S.S.YS..T..S.Y # 50

%1 ...SYSSY..S..T.Y.T.ST...Y.S...Y.SS..S.SS....SS...Y # 100

%1 ......SS.S.SY.SSS.SSSY....S.S.S...S......S........ # 150

%1 ....Y.....Y...S................SS.SS...S...Y.....S # 200

%1 .......Y............S..............S..Y........... # 250

%1 ......S...............S...S....S.................S # 300

%1 ...Y........T...T.......YT...T.........S.....S.... # 350

%1 ................S..T..........................Y... # 400

%1 ......................................S........... # 450

%1 .................................................. # 500

%1 ............S............Y


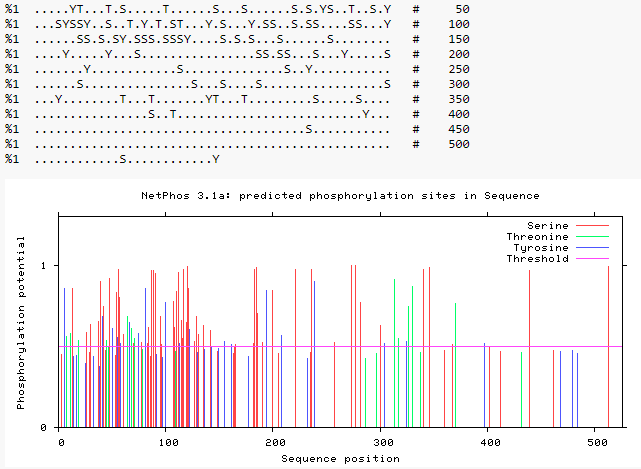


**FUS^R503fs^**：84 phosphorylation sites

%1 .....YT...T.S.....T......S...S......S.S.YS..T..S.Y # 50

%1 ...SYSSY..S..T.Y.T.ST...Y.S...Y.SS..S.SS....SS...Y # 100

%1 ......SS.S.SY.SSS.SSSY....S.S.S...S......S........ # 150

%1 ....Y.....Y...S................SS.SS...S...Y.....S # 200

%1 .......Y............S..............S..Y........... # 250

%1 ......S...............S...S....S.................S # 300

%1 ...Y........T...T.......YT...T.........S.....S.... # 350

%1 ................S..T..........................Y... # 400

%1 ......................................S........... # 450

%1 .................................................. # 500

%1 ...............


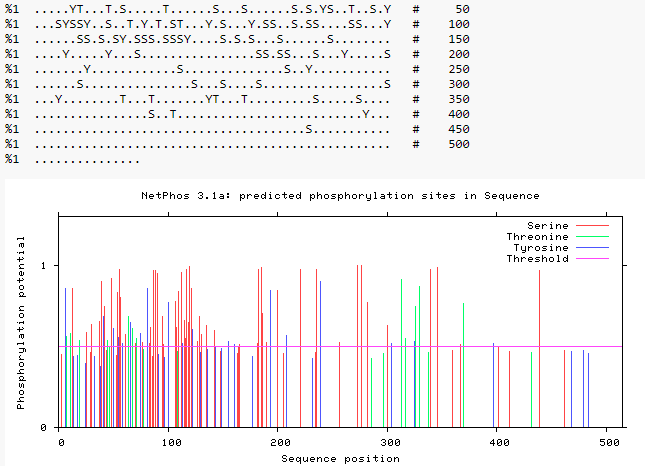


**Sup.5 Hydrophobicity analysis**

The positive number is hydrophobic, and the negative number is hydrophilic. Analysis showed that the protein was mainly hydrophilic before and after mutation.

FUS：Max = 0.756, Min = -3.911


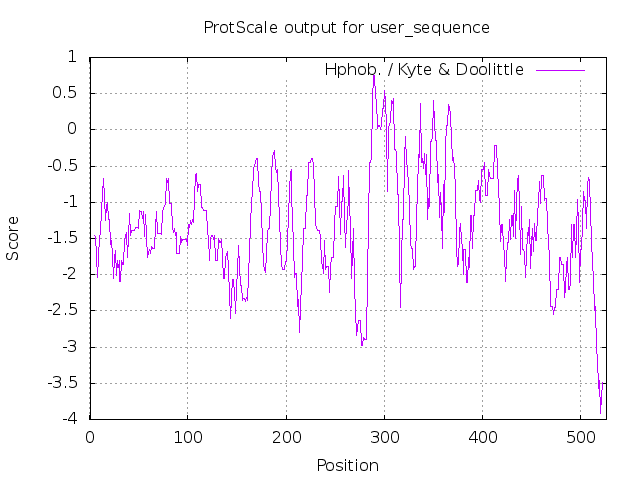


FUS^R503fs^：Max = 0.756, Min = -2.978


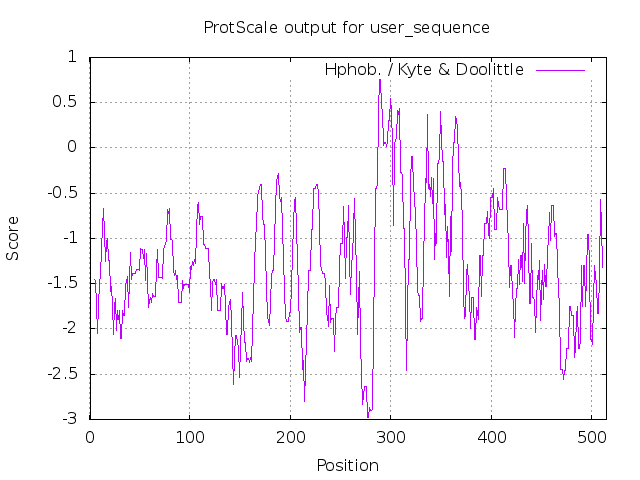


**Difference part:**

| **FUS** | **Score** | **FUS^R503fs^** | **Score** |
| --- | --- | --- | --- |
| 500 | -1.656 | 500 | -2.111 |
| 501 | -1.656 | 501 | -2.167 |
| 502 | -1.300 | 502 | -1.700 |
| 503 | -0.844 | 503 | -1.300 |
| 504 | -0.978 | 504 | -1.433 |
| 505 | -0.978 | 505 | -1.489 |
| 506 | -1.367 | 506 | -1.833 |
| 507 | -0.767 | 507 | -1.833 |
| 508 | -0.656 | 508 | -1.378 |
| 509 | -0.700 | 509 | -0.567 |
| 510 | -1.156 | 510 | -0.856 |
| 511 | -1.511 |  |  |
| 512 | -1.856 |  |  |
| 513 | -2.033 |  |  |
| 514 | -2.489 |  |  |
| 515 | -2.444 |  |  |
| 516 | -3.044 |  |  |
| 517 | -3.156 |  |  |
| 518 | -3.567 |  |  |
| 519 | -3.456 |  |  |
| 520 | -3.911 |  |  |
| 521 | -3.700 |  |  |

**Sup.6 Glycosylation site**

| **O-Glycosylation site** **of FUS** | | **O-Glycosylation site** **of FUS^R503fs^** | |
| --- | --- | --- | --- |
| Number | Name | Number | Name |
| 3 | N | 3 | N |
| 11 | Q | 13 | Y |
| 13 | Y | 29 | S |
| 19 | Q | 54 | Y |
| 29 | S | 57 | Y |
| 54 | Y | 70 | T |
| 57 | Y | 71 | P |
| 70 | T | 107 | S |
| 71 | P | 116 | S |
| 77 | T | 119 | S |
| 87 | Q | 121 | Y |
| 107 | S | 367 | F |
| 116 | S | 412 | G |
| 119 | S |  |  |
| 121 | Y |  |  |
| 221 | G |  |  |
| 257 | D |  |  |
| 367 | F |  |  |
| 412 | G |  |  |
| 462 | H |  |  |
| 513 | Q |  |  |

**N-Glycosylation site (No difference)**


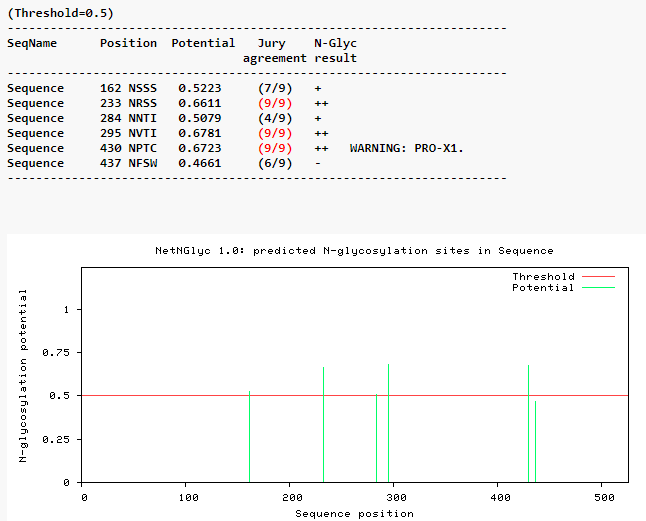


**Sup.7 Differentiation and identification of motor neurons.**


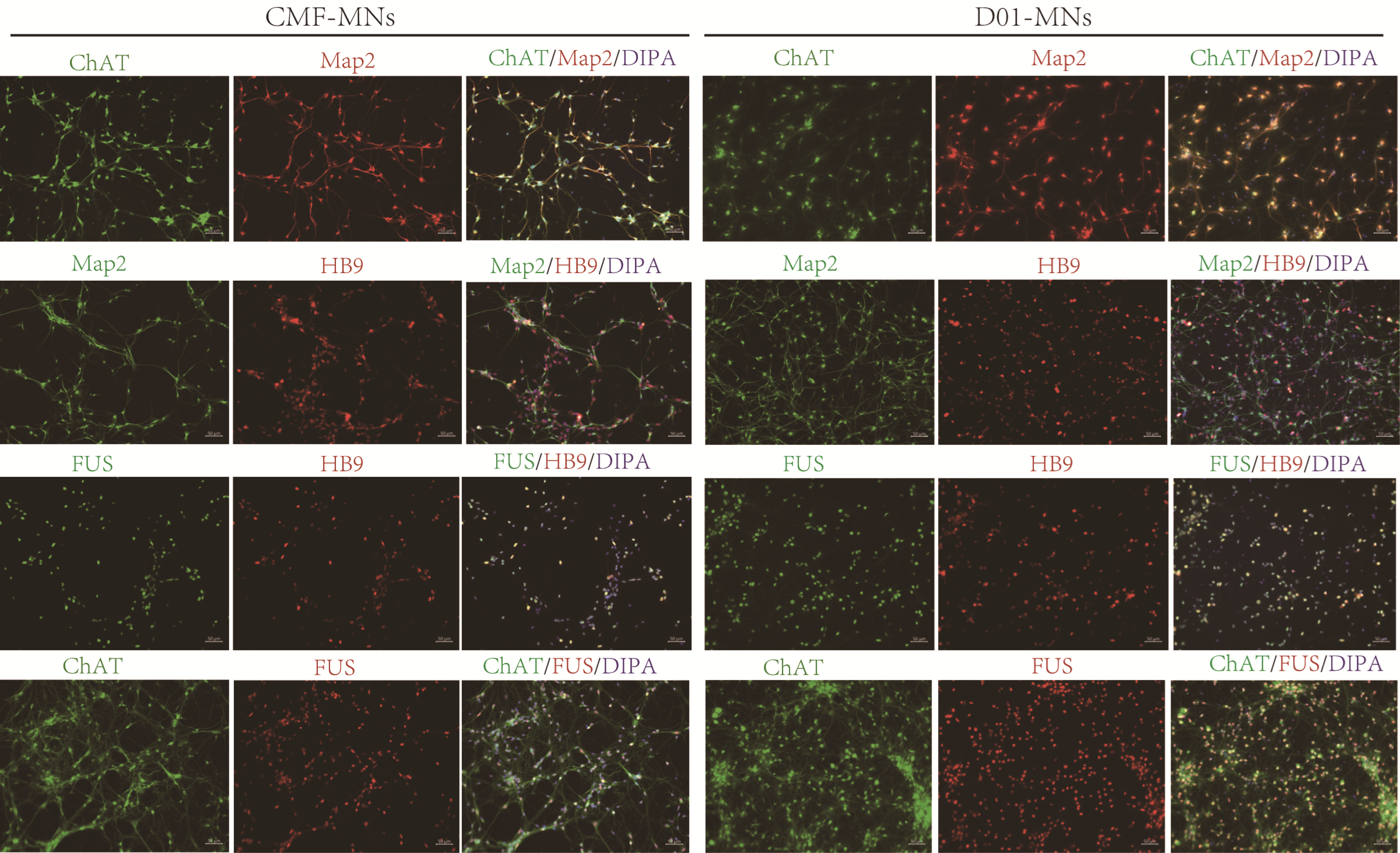


MAP2 is neuron marker; ChAT and HB9 are motor neurons marker; MAP2 and ChAT are cytoplasmic markers; HB9 is a nuclear marker. The proportion of MAP2 and ChAT positive cells was > 95%; HB9-positive cells were 97.27%.
